# On the Relationship between Corneal Biomechanics, Macrostructure, and Optical Properties

**DOI:** 10.3390/jimaging7120280

**Published:** 2021-12-18

**Authors:** Francisco J. Ávila, Maria Concepción Marcellán, Laura Remón

**Affiliations:** Departamento de Física Aplicada, Universidad de Zaragoza, 50009 Zaragoza, Spain; mcvidosa@unizar.es (M.C.M.); lauremar@unizar.es (L.R.)

**Keywords:** corneal biomechanics, corneal structure, corneal aberrations, optical density, Scheimpflug imaging, ocular response analyzer

## Abstract

Optical properties of the cornea are responsible for correct vision; the ultrastructure allows optical transparency, and the biomechanical properties govern the shape, elasticity, or stiffness of the cornea, affecting ocular integrity and intraocular pressure. Therefore, the optical aberrations, corneal transparency, structure, and biomechanics play a fundamental role in the optical quality of human vision, ocular health, and refractive surgery outcomes. However, the inter-relationships of those properties are not yet reported at a macroscopic scale within the hierarchical structure of the cornea. This work explores the relationships between the biomechanics, structure, and optical properties (corneal aberrations and optical density) at a macro-structural level of the cornea through dual Placido–Scheimpflug imaging and air-puff tonometry systems in a healthy young adult population. Results showed correlation between optical transparency, corneal macrostructure, and biomechanics, whereas corneal aberrations and in particular spherical terms remained independent. A compensation mechanism for the spherical aberration is proposed through corneal shape and biomechanics.

## 1. Introduction

Corneal biomechanics is a branch of biomedical sciences that deals with the analysis of the stability of the tissue when an external load or pressure is applied [1,2] or when the intraocular pressure fluctuates. The biomechanical properties of the cornea are responsible for its shape and integrity, acting as a unique convergence point between balanced ductility to preserve aspherical geometry (and correct ocular refraction), stiffness to compensate the intraocular pressure, and an ultrastructure that allows optical transparency. Biomechanical properties of the cornea can be affected by systemic diseases such as diabetes [3,4] or sclerosis [5,6]. In particular, corneal keratoconus may compromise the biomechanical stability, modifying the microstructure [7], weakening mechanical strength [8] and leading to corneal protrusion [9,10], inducing optical aberrations [11,12] that reduce the quality of vision or lead to transplants in advanced stages. The clinical relevance of the study of corneal biomechanics reached special interest with the development of refractive surgery techniques to modify the optical power of the cornea by laser ablation [13] or lenticular extraction [14]. These techniques consist of modifying the lamellar structure of cornea, causing a redistribution of mechanical stress. The biomechanical response is expected to provide the correct corneal curvature [15], and together with optical transparency, normal vision.

On the other hand, corneal transparency has been explained from the hierarchical structure of the cornea. At the molecular scale, X-ray scattering revealed how the collagen ultrastructure within the stroma is responsible for the tridimensional microstructure and consequently for the macroscopic geometry and biomechanics [16].

To date, the maximum spatial resolution of structural hierarchy achieved in living human eyes has been the microscopic scale using two-photon scanning microscopy [17]. However, only confocal microscopes are currently clinically available, and although they allow visualization of the cellular matrix, they are invisible for the stromal architecture [18]. In this sense, Scheimpflug imaging provides excellent tomographic measurements of the macrostructure of the cornea as well as optical density (transparency) [19], which is widely reported in anterior segment analysis for the assessment of normal and keratoconus or ectatic corneas [20] or refractive surgery [21]. Corneal biomechanics is usually assessed employing air-puff tonometry [22]; also, the combination of Scheimpflug imaging and air-puff tonometry has been successfully integrated, bringing excellent results in dynamic assessment of corneal biomechanics [23].

As stated, the molecular organization of the corneal stroma controls the optical transparency, macroscopic shape, and structural stability (biomechanics) [16]. In this work, we will investigate if the relationship between corneal transparency and optical properties, geometry, and biomechanics is preserved at the macroscopic level of the hierarchical structure.

The biomechanical properties, corneal geometry, and optical properties and densitometry measurements were collected from 102 eyes of 51 young-adult healthy subjects using Scheimpflug imaging and air-puff tonometry.

This work focuses on the inter-relationships of corneal biomechanics, optical, and structural properties to bring a comprehensive macroscale characterization of the cornea that can also provide future predictive models of corneal biomechanics.

## 2. Materials and Methods

### 2.1. Participants

This research was reviewed by an independent Ethical Committee of Research of the Health Sciences Institute of Aragon (Spain) approved with reference: C.P.-C.I.PI20/377 (approval date: 14 July 2020). Measurements procedure and data collection were carried out according to the tenets of the Declaration of Helsinki. All participants were informed about the nature, risks, and possible adverse consequences of the study and signed an informed consent document. The ethnicity of the participants involved in this study was European Caucasian, all of them students from the School of Optics and Optometry of the University of Zaragoza (Spain). A total of 102 eyes from 51 healthy young subjects (mean age 24 ± 5) were analyzed using dual Placido–Scheimpflug imaging and air-puff tonometry systems. None of them presented ocular pathologies, corneal disorders, or abnormal intraocular pressure. Exclusion criteria included contact lens wearers due to the influence of contact lens wear in corneal optical density, thickness, and spherical aberration [24].

### 2.2. Experimental Measurements

Clinical measurements were carried out at the laboratory of Optometry of the Department of Applied Physics of the University of Zaragoza and conducted by an experienced clinical optometrist. Both eyes of all participants were analyzed in a sequential procedure: First, optical and geometrical properties (see Table 1) were acquired by a dual Scheimpflug–Placido disk imaging system, and next, corneal biomechanics was assessed using an air-puff tonometer device.

#### 2.2.1. Dual Placido–Scheimpflug Imaging: Structural and Optical Parameters

**Figure 1:**
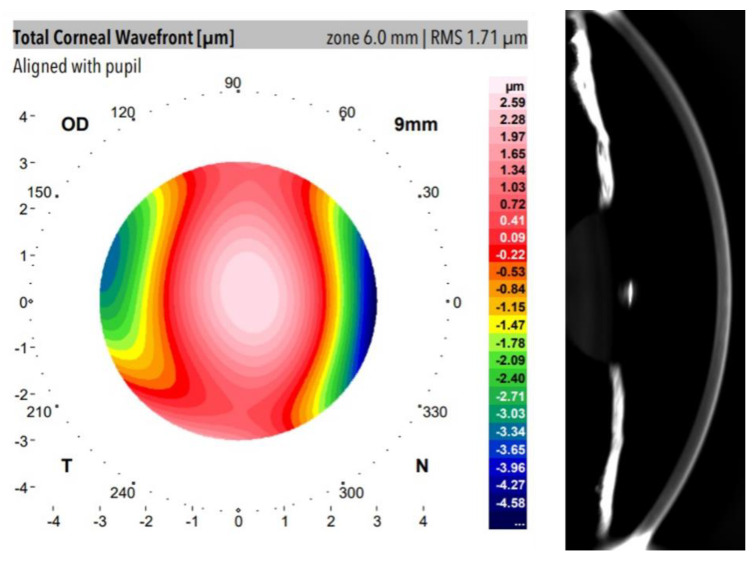
Total corneal wavefront aberration map (**left**) and anterior segment Scheimpflug image (**right**) from a volunteer of the study.

The Galilei Dual Scheimpflug Analyzer (Ziemer Ophthalmic Systems AG, Port, Switzerland) is a clinical optical system that combines Placido Disk imaging and a revolving Scheimpflug camera providing simultaneous acquisition of corneal topography based on the internal and external surfaces including eccentricity, astigmatism, pachymetry (measures of central middle and peripheral cornea), three-dimensional analysis of the cornea, power measures, wavefront aberration, and optical densitometry [25]. Figure 1 shows an example of wavefront aberration mapping and anterior segment Scheimpflug image from a participant of our study. Table 1 summarizes those Galilei outputs selected for data analysis.

##### Optical Transparency Index Calculation

A Galilei device provides the degree of corneal and crystalline lens opacity by computing the relationship between the intensity of the illumination light and back-reflected rays expressed in standarized gray-scale units. It provides macroscopic visualization of the whole anterior segment of the eye by capturing full-angle imaging by rotating the Scheimpflug camera.

Scheimpflug imaging technology visualization is limited through strong scattering or opaque tissues such as the sclera, which is totally opaque due to irregular arrangement of collagen fibrils [16]. Nevertheless, toward the periphery of the iridocorneal angle in Scheimpflug images, a reference gray level from the sclera can be obtained from the Galilei outputs. In this work, the Optical Transparency Index (OTI) is defined and computed as:

(1) $$\mathrm{OTI}=100\times \left[\frac{1}{OD_{Slcera}}\left(OD_{Sclera}-OD_{Cornea}\right)\right].$$

**Figure 2:**
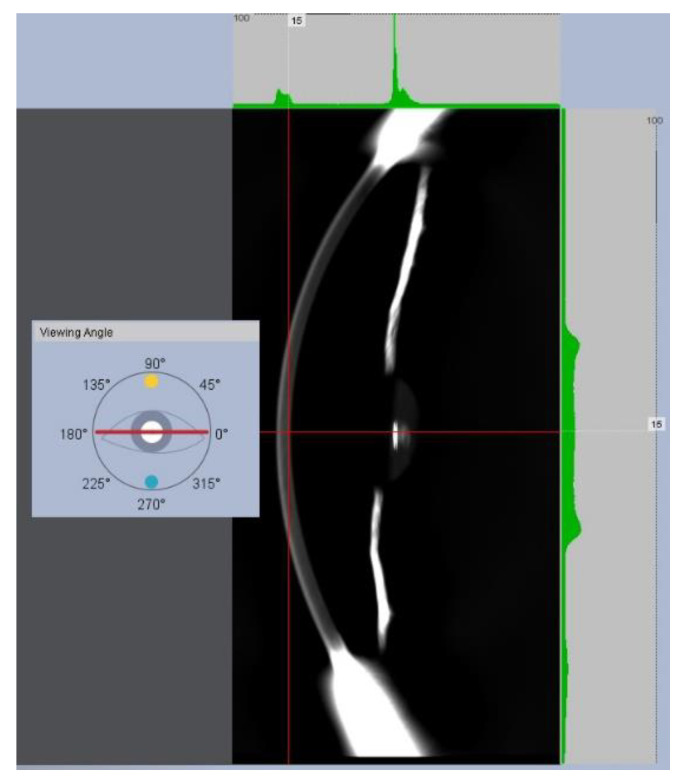
Optical density measurement at the posterior corneal location and horizontal viewing of the Scheimpflug camera.

The OD of the cornea and sclera (*OD_Cornea_* and *OD_Sclera_*) was the average of OD values acquired at the same lateral location corresponding to 4 rotational images acquired at 0°, 45°, 90°, and 135° meridians. Regarding *OD_Cornea_*, the lateral location was the optical axis reference, and the final OD value of each individual frame (i.e., corresponding to a given oriented meridian) was the average of the anterior, central, and posterior axial depths of the cornea. Figure 2 shows an example of an OD measurement at the posterior corneal location at a horizontal viewing angle.

OTI index ranges between 0 (total opacity) and 100 (absence of back-scattering light). The maximum value implies total corneal transmittance for the illumination wavelength underlying the understanding of corneal transparency.

#### 2.2.2. Corneal Biomechanics Assessment

An Ocular Response Analyzer (ORA, Reichert Instruments, Depew, NY, USA) was employed to obtain measurements of corneal biomechanics. ORA is a non-invasive device based on air-puff applanation tonometry that measures intraocular pressure and corneal biomechanics, in particular corneal hysteresis (CH) and corneal resistance factor (CRF) parameters. Briefly, the corneal hysteresis can be defined as the energy dissipation when an external stress is applied, resulting in a time-dependent stain unlike purely elastic materials, which immediately recover their initial state once the stress stops. Whereas CH is a measurement of the viscoelasticity of the cornea, CRF is a measurement of the resistance (i.e., rigidity and/or elasticity) strongly associated to corneal thickness and then an indicator of the corneal pure elastic properties [26]. CRF and CH are related, but they do not describe the same biomechanical properties of the cornea.

**Figure 3:**
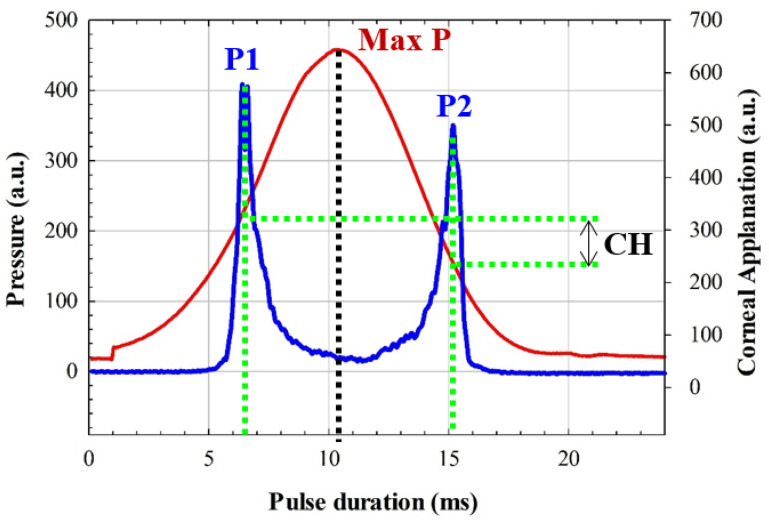
ORA measurement from a participant in our study. P1, P2, Max P, and CH correspond to the first and second applanation, maximum pressure, and corneal hysteresis, respectively.

CRF and CH are computed by quantifying the differential inward and outward corneal responses to an air pulse (see Figure 3) of approximately 24 milliseconds [27]. Once the first applanation is reached, the air pressure causes the cornea to move inward to a slight concavity and then back to a second applanation before recovering the natural shape.

Figure 3 shows an ORA measure from a participant of our study. From pressures P1 and P2 (corresponding to the first and second applanation pressures, respectively), CH and CRF are calculated as [28]:

CH = P1 − P2.(2)

CRF = (P1 − 0.70 × P2) − 3.08.(3)

From an elastodynamic point of view, the first applanation is given when the air pulse compensates for the intraocular and atmospheric pressures. The stress–strain response of the cornea undergoes elastic deformation, whereas the second applanation pressure is affected by energy dissipation; that is, P1 can be related to the pure elastic properties of the cornea, whereas P2 is used for viscoelasticity estimation. For instance, a pure elastic cornea will provide a null CH value and symmetrical pressure curve (see Figure 3) during an ORA measurement, which implies an applanation pressures equalization condition, P1 = P2. In that sense, a stiffness parameter has been reported as the ratio between the resultant pressure at the first applanation and the deflection amplitude [29]. In this study, Equations (1) and (2) were employed to calculate P1 and P2 from ORA outputs (CRF and CH).

#### 2.2.3. Data Analysis

##### Dataset Clustering

Data segmentation is usually applied for dimensionality reduction. In this work, OTI calculations computed from OD measurements made by the Galilei analyzer were found to be discrete values ranging from 78.5 to 81.5 (0.5 step size) for all subjects involved in this study. Table 2 shows the number of subjects (N) that presented a representative OTI value within the range found experimentally, those data are normally distributed (Shapiro–Wilk test), which motivated data clustering for graphical representation and data analysis. Each cluster contains the information of the mean values of each corneal parameter and its standard deviation. In that sense, graphical representation and statistical analysis have been performed on clustered data.

**Figure 4:**
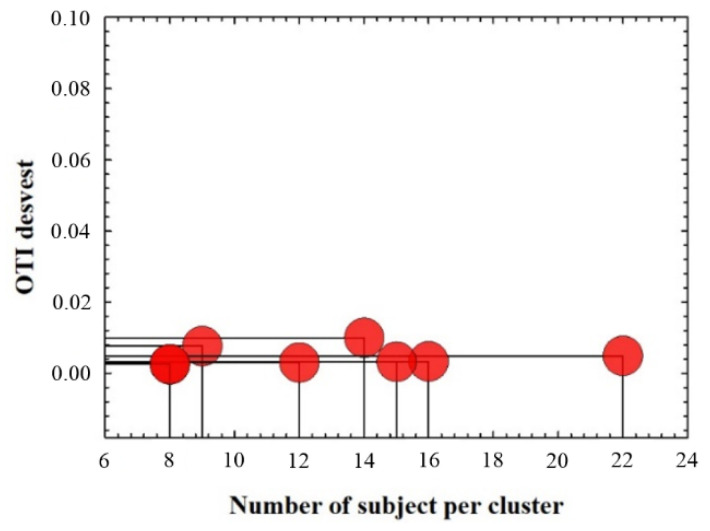
Standard deviation of OTI values as a function of the number of subjects per cluster.

Figure 4 shows the standard deviation of the computed OTI value as a function of the number of subject per discrete OTI value, all of the values were below 1% of error. Therefore, any significant variability in the variance of the clusters will be due to that corneal parameter to be correlated. In that sense, data clustering provides separate visualization of the entire dataset as a function of the meaningful features.

Collected data were stored into an Excel spreadsheet and then migrated to Origin Lab software (Origin Lab Corp., Northampton, MA, USA) for graphical representations and statistical analysis.

Statistical analysis consisted of Spearman Rank Order Correlation and linear regression analysis in order to establish or discard significant relationships between geometrical and optical parameters. The Shapiro–Wilk test was used to test the normality and make valid the inferences of linear regression analysis (power of performed tests = 0.995 with alpha = 0.05). The significance of the linear regression models was also indicated by the F-test. Limits of agreement were used to quantify the agreement between those parameters in graphical representations. Statistics was performed using the advanced statistical tool of Origin Lab software.

## 3. Results

This section shows results of our study that are structured in the analysis of the relationship between optical density of the cornea and three main factors: optical aberrations, biomechanics, and macroscopic geometry of the cornea.

### 3.1. Optical Density and Corneal Aberrations

Figure 1 showed an example of the total aberration wavefont map and the corresponding Scheimpflug image from a participant measured with a dual Galilei imaging system. Total and spherical aberration RMS and OTI average values (mean of 102 eyes measurements) are shown in Table 3. Since spherical aberration is the dominant high-order term at the cornea [30], it was evaluated apart from the total aberration RMS values. The statistical analysis (Spearman correlation) revealed no correlation between optical aberrations (neither total nor spherical term) and OTI values of the cornea. Whereas aberrations govern the correct focus of light and retinal image quality [31], it seems to be unrelated to the transparency of the cornea.

### 3.2. Optical Density and Corneal Macro-Structure

The cornea and sclera are composed mainly of type-I fibrillar collagen whose structural organization at the molecular scale is responsible for optical transparency. Due to the hierarchical structure of the cornea [32], the macroscopic structure is a consequence of the microscopic arrangement. This section investigates if the macroscopic structure of the cornea plays a role in the optical density or it disappears as a corneal transparency factor within the hierarachical organization. The total corneal astigmatism, anterior and posterior eccentricity, central, middle and peripheral thickness were measured and compared with the OTI index for all subjects.

**Figure 5:**
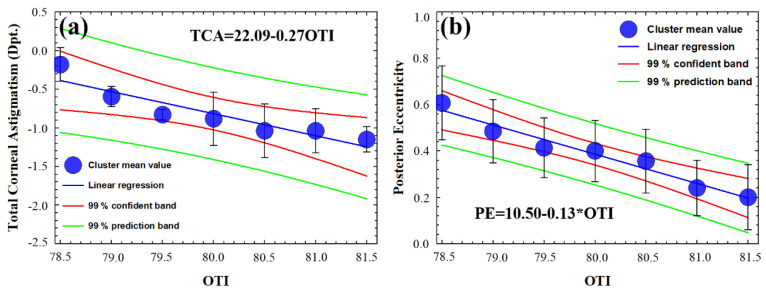
Mean clustered OTI values as function of total corneal astigmatism (TCA) (**a**) and posterior eccentricity (PE) (**b**) for all subjects. Standard deviation of the clustered data, equations, confident, and prediction bands of the regression analysis are included.

Table 4 shows the mean values of thickness, corneal astigmatism, and eccentricity computed from the 102 measured eyes. The statistical analysis revealed that the corneal thickness and optical density are not related in young healthy subjects; however, both total corneal astigmatism and posterior eccentricity (i.e., the inner surface of the cornea) showed strong correlation with OTI parameters. These results imply that in the absence of pathological or physiological (aging) scattering contributions at the cornea, its optical transparency is related to the shape regardless of the thickness. Figure 5 shows the graphical representation of the cluster sampled OTI values as a function of total corneal astigmatism (Figure 5a) and posterior eccentricity (Figure 5b). The statistical analysis revealed positive correlation (Spearman, R^2^ = 0.87) between OTI and total corneal astigmatism (TCA), the regression analysis (overall F-test *p* = 0.003) confirmed that OTI and TCA are related.

Regarding the posterior eccentricity (PE) of the cornea, Figure 5b shows negative correlation (Spearman, R^2^ =0.94) between OTI and PE; the results of the linear regression analysis provided the significant relationship (overall F-test *p* =0.003). However, OTI and anterior eccentricity were found to be independent corneal parameters (see Table 3).

### 3.3. Optical Density and Corneal Biomechanics

**Figure 6:**
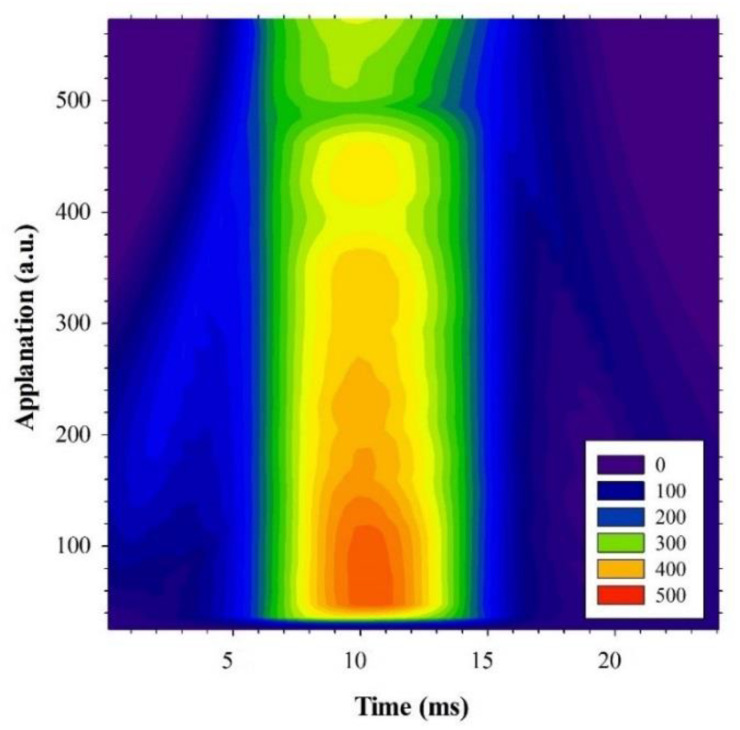
Dynamical representation of corneal applanation as a function of time from a volunteer of the study. Air pulse pressure is scaled in arbitrary units and shown in the bottom right corner legend.

This subsection investigates the impact of biomechanics on corneal transparency. Figure 6 shows a biomechanical image that represents the dynamic stress–strain response of the cornea during an air-puff applanation measurement at ORA device. The maximum deformation occurs at approximately 10 milliseconds. It can be observed how the distribution of the pressure curve is not symmetric but lopsided; this skewness is due to the viscoelastic nature of the cornea, while symmetric distributions correspond to corneas characterized by purely elastic biomechanical properties.

**Figure 7:**
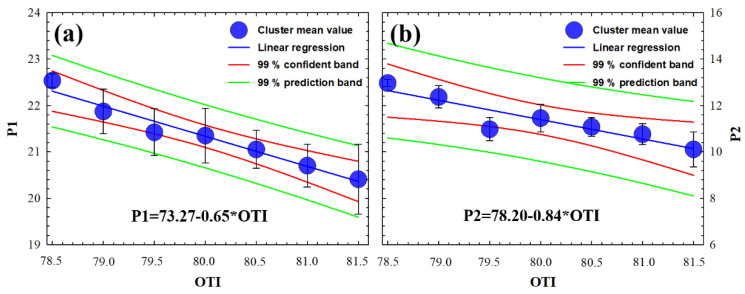
Mean clustered OTI values as function of first (**a**) and second applanation pressures (**b**) at the ORA device for all subjects. Standard deviation of the clustered data, equations, confidence, and prediction bands of the regression analysis are included.

As described in the Methods section, the ORA device provides CH and CRF biomechanical parameters. From them, we derived the applanation pressures P1 and P2, which are related to the pure elastic (P1) and viscoelastic (P2) properties of the cornea. Figure 7 shows the graphical representation of the clustered sampling OTI values as a function of P1 (Figure 7a) and P2 (Figure 7b). The statistical analysis revealed significant (negative) correlations of OTI with P1 and P2 (R^2^ = 0.96 and R^2^ = 0.95, respectively). The error bars that correspond to the standard deviation of the cluster mean value are within the prediction bands of the regression analysis (overall F-tests *p* = 0.001 and *p* = 0.003, respectively). The statistical relationship between P1 and P2 with OTI proves that the optical density of the cornea is related to both elastic and viscosity properties from a macroscopic approach.

### 3.4. Experimental Expression to Predict the Human Corneal Transparency through Macroscopic Parameters

The results above described deal with the separated investigation of the relationship between optical density and corneal parameters that describe the optical, biomechanical, and macro-structural properties of the cornea. In this last subsection, the presence (or lack of) relationships between those characterization parameters are investigated.

**Figure 8:**
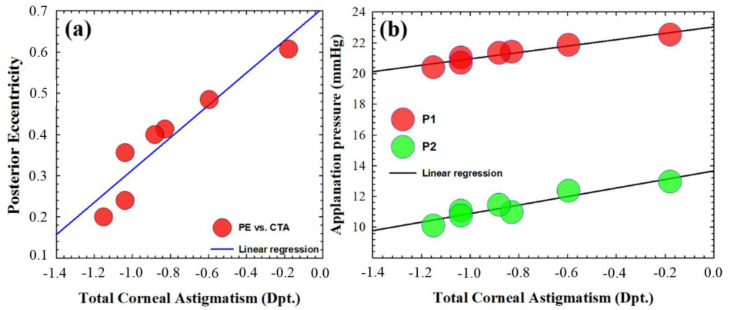
Mean clustered posterior eccentricity versus total corneal astigmatism values (**a**) and mean clustered applanation pressures at ORA versus total corneal astigmatism values (**b**). Linear regression fits are included.

Regarding the macro-structural approach, Figure 8a shows a regression plot of PE versus TCA cluster mean values. The statistical results (R^2^ = 0.88, *p* = 0.0028) revealed that as the total corneal astigmatism decreases, the posterior eccentricity significantly increases.

In this sense, it is obvious that the more direct the influence of the macro-structural shape on cornel aberrometry measurements, the less evident the relationship found between biomechanics and corneal aberrations. However, our findings did not show any statistical relationship in either total or spherical aberration (which is the dominant high-order term in the cornea) with respect to corneal biomechanics in young healthy subjects.

In that sense, corneal astigmatism is one of the low-order aberration terms that plays a fundamental role in ocular refraction; however, in terms of wave-front distortion, it does not increase the total RMS enough to show the dependence of total corneal aberrations with OTI or corneal biomechanics. However, understanding total corneal astigmatism as a measure of corneal asymmetry (structural parameter), Figure 8b shows the linear regression plot of applanation pressures (i.e., P1 and P2) versus total corneal astigmatism. The regression analysis revealed a strong dependence of the required pressures at ORA for first (R^2^ = 0.94, *p* = 0.001) and second ((R^2^ = 0.89, *p* = 0.002) applanations as the corneal astigmatism increases; that is, the higher the corneal asymmetry (measured by astigmatism), the lower the required air pressure to flatten the cornea. It is worth highlighting that the higher correlation value corresponds to the first applanation pressure, which is related to pure elastic properties unlike P2, which is related to viscous properties.

Finally, multiple regression analysis revealed that corneal transparency can be modeled from those macroscopic parameters that showed significant correlation. OTI parameter can be predicted from a linear combination (significant dependence) of the following variables: P1(*p* = 0.13), P2 (*p* = 0.22), TCA (*p* = 0.15), and PE (*p* = 0.021) (analysis of the variance of the multiple linear regression, *p* = 0.018) as:

OTI = 236,895 − [(7.817 × P1) + (0.561 × P2) + (4.458 × TCA) + (18.497 × PE)].(4)

## 4. Discussion and Conclusions

Whereas the role of the corneal ultrastructure in the three-dimensional architecture at microscale, corneal transparency, and mechanical stability is well understood [16], the inter-relationships of those properties at the macroscale within the hierarchical corneal structure has not been investigated.

Considering that most of the medical devices for corneal analysis offer macro-structural resolution and information, in this study, we explore the relationship between the biomechanics, shape, and optical properties of the cornea in healthy young subjects. We analyzed wavefront aberration, optical density, geometrical parameters (thickness, eccentricity, and total corneal astigmatism) and corneal biomechanics using dual Placido–Scheimpflug analyzer and air-puff applanation tonometry systems, respectively.

Corneal aberrations and, in particular the spherical term, seem to have a compensation mechanism to keep stable the retinal image quality in the presence of intraocular scattering [31]; also, in traumatic injuries, corneal opacity appears together with high-order aberrations [33]. In particular, the decrease in optical density and corneal spherical aberration is correlated in contact lens wearers due to corneal swelling [24].

Although corneal astigmatism is one of the most representative low-order aberrations, in our work, we employed the total corneal astigmatism as a measure of the asymmetry in corneal shape (macro-structure parameter) instead of a wavefront distortion, since the contribution of astigmatism aberration term to the total aberration RMS was not enough to establish a relationship between corneal aberrations and OTI.

Regarding structural aspects, the corneal thickness and optical density change in dry eye syndrome, diabetes, and glaucoma [34] are correlated in edema processes such as corneal swelling associated with contact lens wear [24]. However, our findings showed that corneal thickness does not affect the optical transparency in healthy young subjects.

In addition, corneal surface irregularities are associated not only to optical aberrations but also to light scattering [35]. In that sense, our study also included as macro-structural parameters total corneal astigmatism and anterior and posterior eccentricities. As shown in the Results section, the higher the OTI value, the higher the corneal astigmatism and the lower the posterior eccentricity of the cornea. It is worth mentioning that only posterior corneal eccentricity depends on corneal astigmatism [36], and as shown in the Results section, the total corneal astigmatism and posterior corneal eccentricity were negatively correlated (R^2^ = 0.88, *p* = 0.028), which implies that the optical transparency and corneal macrostructure are related by means of the relative shape of the cornea independently of the thickness.

Focusing at the molecular scale, the structure of Type-I corneal collagen is responsible for the optical transparency and three-dimensional arrangement of the stroma [16]. The three-dimensional arrangement of the cornea determines its shape (geometry) and biomechanics [37]. Changes in corneal structure associated to aging or disease factors alter corneal biomechanics [38]. Thus, in our work, we also explored if biomechanics and corneal transparency are actually related at the macroscopic level of study. Results showed (see Figure 6) that both elastic- (P1) and viscoelastic-related parameters (P2) are strongly correlated with OTI; the viscoelastic parameter is more weakly correlated, which implies the dominance of the elastic property in corneal transparency.

Finally, a statistical relationship between biomechanics and corneal geometry (measured by total astigmatism) was found. That is, the corneal astigmatism, elastic, and viscoelastic properties of the cornea are related. It is worth mentioning that the dynamic corneal response depends not only on intrinsic biomechanical properties but also on intraocular pressure and corneal geometry [39]. In that sense, the ORA device provides corrected biomechanical measurements from intraocular pressure and central corneal thickness [23] but does not consider corneal astigmatism as a geometrical parameter affecting biomechanical assessment.

The relationship between corneal aberrations (in particular spherical term) and biomechanics has been previously stated in keratoconic eyes [40]. However, in young healthy subjects, the spherical aberration and corneal biomechanics are not related.

The independence of spherical aberration of corneal biomechanics in young healthy subjects could be explained by a feedback cycle compensation mechanism that occurs in the posterior cornea considering that biomechanical measurements are carried out at the anterior surface of the cornea: our results showed on the one hand that posterior eccentricity significantly increases as total astigmatism decreases, and on the other hand, the lower the total astigmatism of the cornea, the higher the applanation pressures at ORA (i.e., P1 and P2).

These results are consistent with the study reported by Li et al. [41], since their work concluded that the posterior corneal surface plays an important role in compensating for spherical aberration of the anterior corneal surface.

To conclude, within the hierarchical structure of the cornea, the nanoscopic scale leads to optical transparency, whereas the microscopic architecture models corneal biomechanics that impact the macroscale [16]. Previously to our work, Garzón et al. [42] reported a study on corneal densitometry and its correlation with aging, corneal thickness and curvature, and refractive error using Scheimpflug imaging; they found no correlation between optical transparency and refractive parameters. In our study, we expanded the analysis of those factors that could have an impact on corneal transparency.

In conclusion, if the macroscopic structure of the cornea is connected through optical and geometrical properties, its optical transparency can be modelled. Optical transparency measured through macroscale approaches is related to corneal biomechanics; in particular, the elastic property seems to be the dominant contribution. In addition, corneal astigmatism affects the biomechanical measurements in the sense that less applanation pressure is required as the total astigmatism increases, so future corrections must be calibrated into air-puff measurements to establish a compensation for asymmetric corneas and in short, avoid underestimation of the corneal biomechanics assessment.

Future research will expand this dataset to be incorporated into a predictive convolutional neural network including aging and pathological conditions to help us to develop predictive models in terms of the inter-relationships of the optical properties, structure, and biomechanics of the cornea.

## Figures and Tables

**Table 1:** Structural and optical parameters from the Galilei system considered of interest in our study. The total and spherical aberration wavefront were numerically evaluated by its root mean square (RMS) value.

Structural Parameters	Optical Parameters
Central Thickness	Total Aberration RMS
Middle Thickness	Spherical Aberration RMS
Peripheral Thickness	
Anterior Eccentricity	
Posterior Eccentricity	Optical Density
Total Corneal Astigmatism	

**Table 2:** Clustering of the OTI values as a function of the number of subjects (N).

OTI	N	Cluster
78.5	14	1
79.0	9	2
79.5	22	3
80.0	16	4
80.5	15	5
81.0	12	6
81.5	8	7

**Table 3:** Optical transparency index (OTI); Total aberration RMS (TA RMS); Spherical aberration RMS (SA RMS); and Spearman correlation analysis for the data collected from all participants of the study (* *p* < 0.05); ** *p* < 0.005).

OTI	TA RMS * (µm)	SA RMS ** (µm)	Spearman’s *	Spearman’s **
80.0 ± 0.9	1.45 ± 0.28	−0.15 ± 0.05	Failed, *p* = 0.38.	Failed, *p* = 0.26.

**Table 4:** Mean values of geometrical parameters and correlation results with OTI index.

Structural Parameter	Location	Mean Value	Spearman’s
Corneal Thickness	Central	554 ± 30 µm	Failed, *p* = 0.58.
	Middle	601 ± 30 µm	Failed, *p* = 0.42.
	Peripheral	674 ± 69 µm	Failed, *p* = 0.29.
Corneal Astigmatism	Global	0.87 ± 0.34 Dpt.	R^2^ = 0.87
		
Anterior Eccentricity	Global	0.24 ± 0.16	Failed, *p* = 0.39
Posterior Eccentricity	Global	0.42 ± 0.12	R^2^ = 0.94
